# Preimplantation Genetic Testing for Aneuploidy Improves Live Birth Rates with In Vitro Produced Bovine Embryos: A Blind Retrospective Study

**DOI:** 10.3390/cells10092284

**Published:** 2021-09-02

**Authors:** Giuseppe Silvestri, Carla Canedo-Ribeiro, María Serrano-Albal, Remi Labrecque, Patrick Blondin, Steven G. Larmer, Gabriele Marras, Desmond A.R. Tutt, Alan H. Handyside, Marta Farré, Kevin D. Sinclair, Darren K. Griffin

**Affiliations:** 1 School of Biosciences, University of Kent, Canterbury CT2 7NH, UK; G.Silvestri@kent.ac.uk (G.S.); cfcr2@kent.ac.uk (C.C.-R.); ms2104@kent.ac.uk (M.S.-A.); alan.handyside@outlook.com (A.H.H.); m.farre-belmonte@kent.ac.uk (M.F.); 2 L’Alliance Boviteq Inc., Saint-Hyacinthe, QC J2T 5H1, Canada; rlabrecque@boviteq.com (R.L.); pblondin@boviteq.com (P.B.); slarmer@semex.com (S.G.L.); gmarras@semex.com (G.M.); 3 School of Biosciences, University of Nottingham, Nottingham LE12 5RD, UK; des.tutt@nottingham.ac.uk (D.A.R.T.); kevin.sinclair@nottingham.ac.uk (K.D.S.)

**Keywords:** cattle, breeding, single nucleotide polymorphism (SNP), mosaicism

## Abstract

Approximately one million in vitro produced (IVP) cattle embryos are transferred worldwide each year as a way to improve the rates of genetic gain. The most advanced programmes also apply genomic selection at the embryonic stage by SNP genotyping and the calculation of genomic estimated breeding values (GEBVs). However, a high proportion of cattle embryos fail to establish a pregnancy. Here, we demonstrate that further interrogation of the SNP data collected for GEBVs can effectively remove aneuploid embryos from the pool, improving live births per embryo transfer (ET). Using three preimplantation genetic testing for aneuploidy (PGT-A) approaches, we assessed 1713 cattle blastocysts in a blind, retrospective analysis. Our findings indicate aneuploid embryos have a 5.8% chance of establishing a pregnancy and a 5.0% chance of given rise to a live birth. This compares to 59.6% and 46.7% for euploid embryos (*p* < 0.0001). PGT-A improved overall pregnancy and live birth rates by 7.5% and 5.8%, respectively (*p* < 0.0001). More detailed analyses revealed donor, chromosome, stage, grade, and sex-specific rates of error. Notably, we discovered a significantly higher incidence of aneuploidy in XY embryos and, as in humans, detected a preponderance of maternal meiosis I errors. Our data strongly support the use of PGT-A in cattle IVP programmes.

## 1. Introduction

After humans (and possibly mice), the most studied mammalian species at the preimplantation stage of development (1–7 days post-fertilization) is cattle. In vitro production (IVP: the cattle equivalent of human IVF) techniques are used extensively for breeding purposes in order to shorten generation intervals and facilitate the bio-secure transportation of genetic resources [1]. The high demand for dairy and beef products has spurred the implementation of new approaches in IVP, combining the most recent advances in embryology with genomics and bioinformatics. Currently, around one million IVP cattle embryos are transferred annually, with a growing proportion screened for genomic estimated breeding values (GEBVs) before embryo transfer (ET) [2,3]. The idea of employing a genomic evaluation to estimate breeding values of individual animals is not new [4]. The in-depth characterisation of the bovine genome sequence has accelerated this trend, and has allowed the formulation of accurate models that describe the association between certain genetic markers (often SNPs: single nucleotide polymorphisms) and valuable traits. GEBVs have traditionally been determined soon after birth; however, modern screening at the embryonic stage has the advantage of shortening generation times further (to facilitate more rapid introduction of new genetics) and increasing selection intensity (by increasing the pool of screened individuals). In brief, the approach involves four stages: the biopsy of a portion of the trophectoderm, whole genome amplification (WGA), interrogation of a SNP array, and the subsequent bioinformatics analysis. The embryo transfer then proceeds as with any other IVP process. Currently in IVP, however, the proportion of live births per ET is still only around 50%, depending on embryo quality and the production system employed (for a comprehensive review, see [5]). Moreover, there are no known GEBVs yet discovered that predict the chances of embryo implantation.

The presence of extra or missing chromosomes (principally aneuploidy) causes IVF failure in humans. Aneuploidy can originate during meiosis in either gamete (predominantly the oocyte) or in the embryo itself during the mitotic divisions of preimplantation development. The latter can give rise to mosaicism: a mixture of euploid and abnormal cells. Preimplantation genetic testing for aneuploidy (PGT-A), the practice of screening for chromosomal abnormalities prior to ET, has been the focus of much debate in the medical literature. Whilst the fact that there is a higher viability of euploid embryos seems to be well established, a recent randomised controlled trial gave mixed results, showing no overall benefit for PGT-A, except in older age groups [6]. This apparent paradox could be explained by a negative impact on embryo viability of the biopsy procedures (a pre-requisite for established PGT-A methods) or by misdiagnoses leading to embryo wastage [7]. Indeed, many cohort and retrospective studies have suggested that there is indeed a benefit of performing PGT-A [8].

Between 25 and 40% of cattle embryos produced by IVP carry a chromosomal abnormality [9,10,11], which (as in humans) can lead to early embryo development failure, pregnancy loss, or the birth of infertile animals. However, the use of PGT-A is not well established in animal breeding, and it has only recently been adapted to cattle screening, showing promising results [10]. Interrogating the SNP chip data to combine GEBV with PGT-A offers breeders the potential to achieve better embryo selection, and attain a higher chance of returning a pregnancy per embryo transferred.

Here, we made use of three methods of deriving aneuploidy information from SNP genotyped embryo biopsy material (discussed hereafter). The signal log R ratio (LRR) and B allele frequency (BAF) were calculated from the SNP intensity data to provide an accurate measure of the chromosome copy number [12]. Karyomapping [13] was employed to trace the parental origin of aneuploidy and the position of recombination events, as well as to diagnose those abnormalities that result in a normal karyotype, as is the case of the uniparental disomy (UPD). Finally, the meiotic or mitotic origin of trisomies was clarified by employing what we will term the Gabriel–Griffin algorithm [14]. 

Despite previous promising results [10,11], the full benefit of PGT-A in cattle breeding has yet to be established. With this in mind, the purpose of this study was to test the hypothesis that a PGT-A strategy involving SNP chip interrogation significantly improves pregnancy and live birth rates for cattle IVP. The possible ramifications for human PGT-A are discussed. 

## 2. Methods

### 2.1. Study Design

All data were acquired by L’Alliance Boviteq Inc. (Saint-Hyacinthe, QC, Canada) as part of their business operations. Ovarian stimulation, IVP, embryo biopsy, WGA, SNP genotyping, and embryo transfer were performed in a controlled commercial environment between 2016 and 2018. The resulting database compiled over this time consisted of 1737 transferred embryos and their 241 parents (168 dams and 73 sires), all of which were genotyped on a variety of different Illumina SNP chips as summarised in Table 1. Additionally, L’Alliance Boviteq Inc. kept records of parental age, and of embryo stage and grade, together with pregnancy outcomes following transfer.

The present study was conceived as a retrospective analysis of this database. The PGT-A investigation included n = 1713 embryos derived from n = 73 Holstein sires and n = 168 Holstein dams; 24 embryos were excluded from analysis as they lacked at least one full sibling or two half-siblings (which is a requirement for Karyomapping analysis). The PGT-A operators were blinded with regards to all information pertaining to parental age and history, as well as to any information related to embryo quality, stage, and outcomes following ET. This information was centrally retained by L’Alliance Boviteq Inc., and only disclosed after PGT-A analysis.

### 2.2. Ovarian Stimulation and In Vitro Embryo Production

Ovarian stimulation and IVP were carried out as described previously [15]. Briefly, 48 h following dominant follicle removal, dams underwent ovarian stimulation for 3 days, comprising six intra-muscular injections of FSH (Folltropin-V, Bioniche Animal Health, Belleville, ON, Canada) given at 12 h intervals, followed by a period of FSH withdrawal (coasting) typically lasting around 44 h. Each injection contained 30 to 40 mg of FSH (equivalent to 87.5 to 101.4 IU) at a manufacturer recommended concentration of 20 mg/mL. Cumulus oocyte complexes (COCs) were recovered by ultrasound-guided follicular aspiration in warm HEPES-buffered Tyrode’s medium with heparin (10 IU/mL). In vitro maturation (IVM) was carried out in HEPES-buffered media as previously described [10]. Maturing COCs were incubated for 24 h at 38.5 °C in atmospheric CO_2_.

In vitro fertilisation was undertaken in 40 μL droplets of modified Tyrode’s lactate medium supplemented with 6 mg/mL BSA, 0.2 mM pyruvate, 2 μg/mL heparin, 50 µg/mL gentamycin, and PHE [16]. The culture vessel was a Nunclon 35 mm cell culture dish (Nalgene Nunc, Rochester, NY, USA). Frozen spermatozoa were thawed and selected by discontinuous gradient centrifugation (BoviPure, Nidacon, Göthenborg, Sweden); the final sperm concentration in the fertilisation drops was 1 × 10^6^ cells/mL and the gametes were co-incubated at 38.5 °C for 18 to 22 h under 5.5% CO_2_. The resulting embryos were cultured in Nunclon dishes, using 50 μL droplets of modified SOF medium supplemented with nonessential amino acids, 3 µM EDTA, and 4 mg/mL BSA at 38.5 °C under 6.5% CO_2_ and 5% O_2_.

### 2.3. Embryo Biopsy, WGA, SNP Genotyping, and ET

Embryos were evaluated as per the guidelines presented in [17]. Embryos of developmental stages 4 to 9 of either “Excellent” or “Good” morphology (grades 1 and 2) were washed and held in ViGRO flush solution (EGG Tech, Pullman, WA, USA), and approximatively 15 trophectoderm cells were biopsied using a micro-blade (Feather, Osaka, Japan). The biopsied cells were immediately subjected to whole genome amplification (WGA), which was carried out using a REPLI-g mini kit (Qiagen, Mississauga, ON, Canada) following the manufacturer’s instructions. The resulting WGA products were then submitted for genotyping to a commercial service provider (GeneSeek (Neogen), Lincoln, NE, USA). The biopsied embryos were cryopreserved as previously described [10] before being transferred to suitably prepared recipient cows [18]. Clinical pregnancies were established at 60 days post ET by ultrasound. Hair samples from the parents were collected and sent directly to GeneSeek for a genotyping analysis.

### 2.4. Computing and PGT-A

The SNP genotyping output files were archived by L’Alliance Boviteq Inc. The SNP database was then transferred to the University of Kent for a retrospective PGT-A analysis. Basic data manipulations on the database (cases extraction, file preparation for downstream analysis, filtering for shared SNPs) were performed using custom AWK and BASH scripts and resulted in the creation of output files for the PGT-A analysis. Each embryo included in the final study was analysed using a combination of three PGT-A algorithms, namely: signal intensity data LRR and BAF [12], Karyomapping [10,13], and Gabriel–Griffin plots [14]. Further details as relevant to each of these methods are presented below.

To detect the presence or absence of chromosome imbalances, LRR and BAF graphs were used, (discussed in [19]). The analysis pertaining to LRR and BAF was conducted in R [20] and figures were produced using the package karyoploteR [21]. Average R and Theta values for each SNP combination (R_AA_, R_AB_, R_BB_, Theta_AA_, Theta_AB_, Theta_BB_) were calculated from raw signal intensity data (X and Y) available from the SNP database itself; these values were then used to calculate the relevant data points on each graph (LRR and BAF) following the methodology described by Sun et al. [22]. For aneuploid cases, mosaicism diagnoses at the whole chromosome level were inferred as performed in Tutt et al. [11]. In segmental aneuploidy, only the affected part of the chromosome was used to calculate the mosaicism level for that aneuploidy.

To clarify the origin of each aneuploidy, two approaches were employed [13,14] using in house VBA based algorithms. Output files containing the appropriate information for each sample (target embryo and its parents) were processed through the VBA macro BoVision (version 3.1, University of Kent, updated from [10]), and the VBA macro BoVisionGG (version 1.0, University of Kent).

### 2.5. Limitations

Whole chromosome and segmental errors were determined for all the samples in the study. However, chromosome Y errors could not reliably be investigated due to the limited number of SNPs available for this chromosome and the impossibility to implement Karyomapping’s haploblock tracing. Similarly, paternal chromosome X errors could not be investigated by Karyomapping (sex chromosomes are present in single copy in the sire; therefore, haploblock tracing is prevented).

### 2.6. Statistical Analysis

The statistical analysis was completed on SPSS (version 26, IBM). Binomial data were compared by fitting a binomial or a multinomial generalised linear model with logit link functions. Multiple comparisons were performed using the Bonferroni correction. Continuous variables were investigated by ANOVA or a linear regression analysis as appropriate. The threshold for significance was set as *p* = 0.05. Percentages were reported with their 95% confidence intervals (CI) for proportions. Pregnancy checks and live birth checks were performed for all the embryos transferred in this study (n = 1713). Birth weight checks were performed on n = 656 out of a total n = 700 live borne calves.

### 2.7. Ethical Statement

This study was performed entirely in silico by accessing information archived by L’Alliance Boviteq Inc. as a routine part of their business operations. As such, no live animals or embryos were employed.

### 2.8. Data Availability Statement

The SNP database analysed in this study is the intellectual property of L’Alliance Boviteq Inc.

## 3. Results

### 3.1. PGT-A Improves Pregnancy and Live Birth Outcomes

The levels of aneuploidy were determined by LRR/BAF plots and Karyomapping which, when combined, discovered a chromosomal abnormality in 14.1% (n = 241/1713) of the embryos. When ET outcomes were analysed, it was found that the embryos identified as euploid resulted in a pregnancy rate of 59.6% (n = 878/1472) and a live birth rate of 46.7% (n = 688/1472); in contrast, the embryos diagnosed as chromosomally abnormal resulted in a pregnancy rate of just 5.8% (n = 14/241), and a live birth rate of 5.0% (n = 12/241). In both cases, the difference was statistically significant (*p* < 0.0001). The associated odds ratios (OR) were 24.0 (95% CI: 13.8–41.5) and 16.7 (95% CI: 9.3–30.2) in favour of euploid embryos for pregnancy and live birth rates, respectively. The positive predictive value (PPV) for the type of PGT-A test we performed for live birth rates was therefore 98.3% (95% CI: 97.1–99.0%).

**Figure 1:**
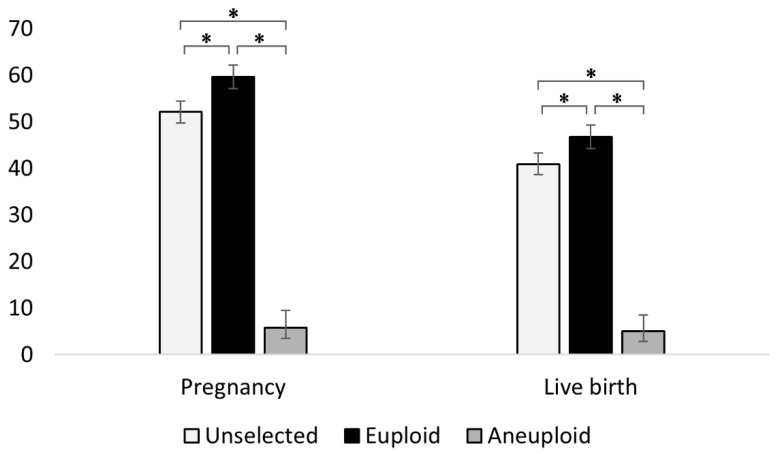
Effect of ploidy on pregnancy and live birth outcomes. Embryos diagnosed as euploid showed higher pregnancy and live birth rates as compared to embryos diagnosed as chromosomally abnormal or indeed to embryos not undergoing PGT-A selection. Sample population consisted of n = 1713 embryo transfers, which resulted in n = 892 pregnancies (unselected group); among these, retrospective PGT-A analysis discovered n = 878 pregnancies from n = 1472 euploid embryos and just n = 14 pregnancies from n = 241 aneuploid embryos. Similarly, there were a total of n = 700 live births (unselected group), which were found to be derived from n = 688 euploid embryos and n = 12 aneuploid embryos. Data presented as percentage (%) with 95% CI. * denotes statistically significant differences (*p* < 0.01).

In the original database, the transfer of embryos without performing PGT-A led to a pregnancy rate of 52.1% (n = 892/1713) and a live birth rate of 40.9% (n = 700/1713), suggesting that the elective transfer of PGT-A selected euploid embryos would have improved pregnancy rates by 7.5% (*p* < 0.0001, OR = 1.36) and live birth rates by 5.8% (*p* =0.001, OR = 1.27). Importantly, from a breeder’s perspective, the exclusive use of embryos diagnosed as euploid would have decreased the number of ETs required to obtain a live birth from 2.45 ETs/livebirth to 2.14 ETs/livebirth. A visual summary of these findings is given in Figure 1.

The proportion of euploid embryos that successfully established a clinical pregnancy at D60 post transfer and then proceeded to yield a live birth was 78.4% (n = 688/878); conversely, among the few aneuploid embryos which also resulted in clinical pregnancies at D60, the live birth rate was 85.7% (n = 12/14). The difference between these two proportions was not statistically significant (*p* = 0.51). Since no data were available on miscarriages within the first few weeks, we were not able to discriminate between early pregnancy loss (before D60) and embryo mortality. Nevertheless, with the limitations imposed by the small number of aneuploid embryos surviving in utero after D60, the data presented seem to suggest the presence of a bottleneck for aneuploid embryos positioned before D60 (be this either an early miscarriage or an embryo attachment failure), with the aneuploid embryos surviving this threshold behaving indistinguishably from the euploid embryos in terms of their ability to proceed to term.

### 3.2. Chromosomal Abnormality Classes: Maternal Meiotic Errors Are by Far the Most Frequent

The combination of PGT-A algorithms employed identified a range of abnormalities including whole chromosome and segmental errors and pinpointed their origin as being either meiotic or mitotic. Further to this, for all meiotic errors, we were able to detect whether the error originated in the maternal or paternal gamete. Additionally, for trisomies, we were also able to pinpoint the precise origin of the chromosomal error (meiosis I, meiosis II, mitosis) by Gabriel–Griffin plots. For monosomies, however, the same approach was unable to indicate whether the error originated in meiosis or during embryo development; instead, monosomies detected just in LRR/BAF plots but not by Karyomapping were classified as having originated de novo in the embryo (and therefore as being of mitotic origin). Overall, the most common abnormality class detected was monosomy, which appeared in 47.6% (n = 139/292) of the errors. In this database, trisomies were slightly underrepresented as compared to monosomies, appearing in 38.7% (n = 113/292) of the errors (*p* = 0.10). The remaining abnormality classes detected included segmental errors, triploidy/hypotriploidy, and uniparental disomy (UPD). These findings are presented in Table 2.

Interestingly, the vast majority of errors for which a parental origin could be determined originated in the oocyte (75.3%, n = 220/292). More specifically, the trisomy analysis by the Gabriel–Griffin plots (for which a full breakdown is given in Supplementary Table S1) revealed that errors occurring during maternal meiosis I accounted for 73.5% of all trisomy events (n = 83/113), suggesting that a similar proportion of monosomies could have had the same origin. The only aneuploidy classes whose origins could be more prevalently ascribed to the paternal germline were triploidy/hypotriploidy (possibly resulting from polyspermy as well as or in place of meiotic errors), and segmental errors.

### 3.3. Mosaicism Incidence, Pregnancy, and Live Birth Outcomes in Chromosomally Abnormal Embryos

Among aneuploid embryos, at least 25.3% (n = 61/241) carried one or more chromosomal abnormalities in a mosaic configuration. A detailed categorisation of these embryos, including their calculated mosaicism percentages is presented in Supplementary Table S2. Only 6.6% of ETs involving at least one mosaic error resulted in a live birth (n = 4/61); similarly, the live birth proportion among aneuploid non-mosaic cases was 4.5% (n = 8/179) and the difference between the two was not significant (*p* = 0.521). The average birth weight of calves born from mosaic and non-mosaic aneuploid cases (average of 42.7 kg and 38.9 kg, respectively) was also not significantly different (*p* = 0.377), although the low sample size of these two groups may be masking any underlying effects. Additionally, the analysis is complicated by the fact that, on the field, parturition may be induced up to 10 days before the actual due date. With these limitations in mind, it is nevertheless interesting to note that the average birth weight did not differ between euploid and chromosomally abnormal embryos (*p* = 0.765). A detailed overview of the chromosomally abnormal embryos which still resulted in live births, is provided in Table 3. We were unfortunately unable to perform a follow-up study of these animals to confirm their ploidy status after birth.

### 3.4. Parental Effects on Aneuploidy Incidence

**Figure 2:**
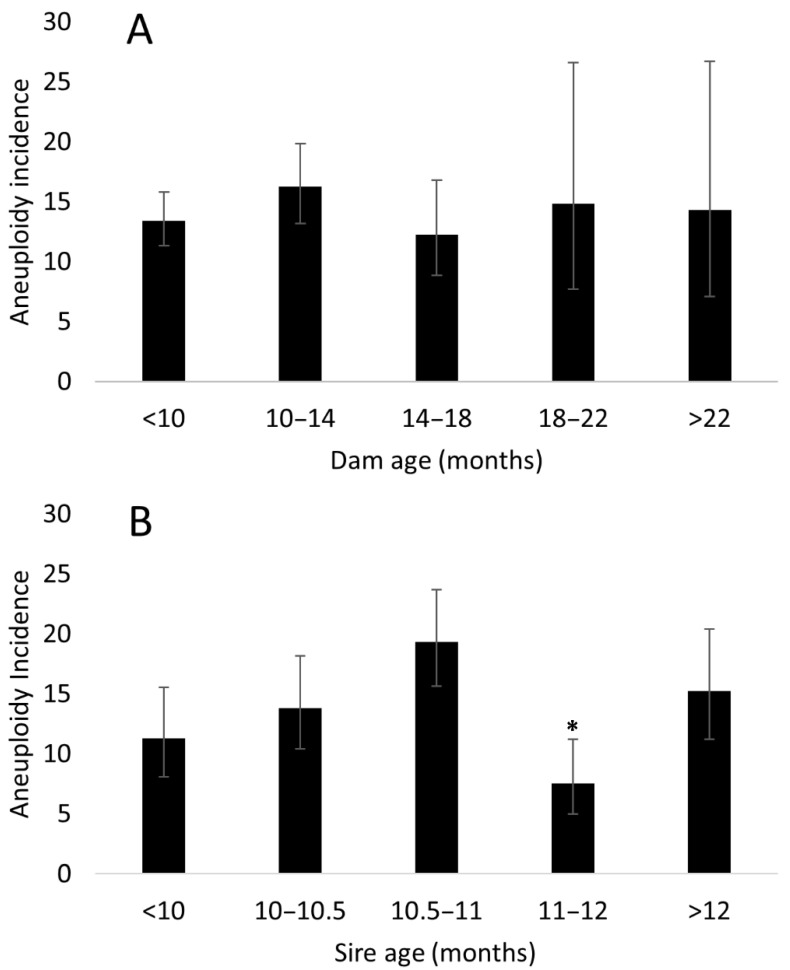
Embryo aneuploidy incidence by parent age. (**A**) Regression analysis showed no significant interaction between the age of the dam at oocyte collection and aneuploidy incidence in the resulting embryos (R^2^ = 0.0142, *p* > 0.05). (**B**) Employing older sires did not negatively affect embryo ploidy; the star symbol (*) denotes a statistically significant (*p* < 0.01) aneuploidy incidence decrease for that group as compared to all others. Data presented as percentage (%) with 95% CI.

The average age of the donors (n = 168) was 10.3 ± 0.7 months. Sire age (n = 63) was recorded in groups (<10 months, 10–10.5 months, 10.5–11 months, 11–12 months, >12 months), with most males collected at either 10.5–11 months (n = 9) or past 12 months of age (n = 12). The specific parent (dam or sire) employed in the IVP cycle seemed to influence the aneuploidy rate. Certain dams were more prone to produce aneuploid embryos (*p* = 0.0002); however, a sire specific effect was not discovered (*p* = 0.636). On the other hand, the age of the donor dam did not seem to have an effect on aneuploidy incidence (*p* = 0.678), a result which may not be surprising given the young average age of the dams employed. Curiously, we discovered a significant effect of sire age on aneuploidy incidence (*p* = 0.002), with a post-hoc investigation suggesting this result is due to a significant decrease in aneuploidy incidence associated with sperm collections performed when the sires were between 11 and 12 months of age. These observations are reported in Figure 2. Nonetheless, it should be noted that the relatively young age of all the parents employed prevents a comprehensive study of parental age effect on the incidence of aneuploidy as relevant to the wider breeding population.

### 3.5. Relationship between Embryo Stage and Grade with Aneuploidy and Pregnancy/Live Birth Rates

**Figure 3:**
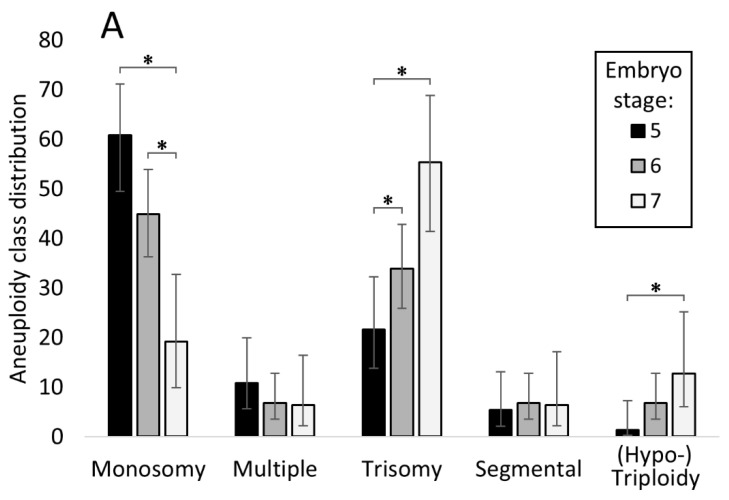

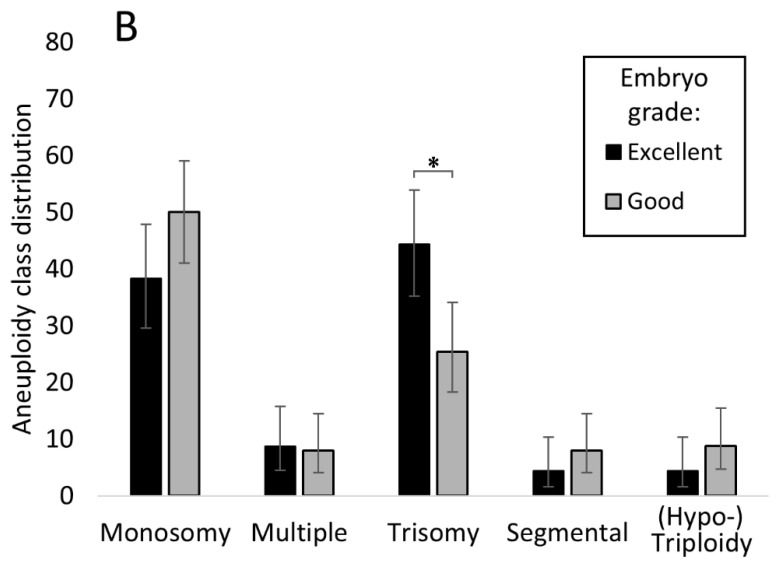
The distribution of aneuploidy types in embryos is affected by developmental stage and morphology grade. (**A**) The proportion of each aneuploidy class changes significantly across different developmental stages, with monosomy being the prevalent form at stage 5 and trisomy at stage 7. Embryos of stages 4, 8, and 9 were omitted from this graph since samples size was less than three in each group. (**B**) Overall aneuploidy class distribution also differed between embryos of “Excellent” or “Good” morphology. Data presented as percentage (%) with 95% CI. * denotes statistically significant differences (*p* < 0.01).

Interestingly, the overall aneuploidy incidence was significantly affected by the embryo’s developmental stage at biopsy (*p* < 0.0001), with more advanced embryos displaying lower rates of chromosomal errors. This trend was also reflected in pregnancy and live birth outcomes, which tended to favour the embryos of later stages (Table 4). A more detailed analysis also revealed that the incidence of specific aneuploidy classes varied considerably within different embryo development stages, as reported in Figure 3A. In particular, the incidence of whole chromosome errors followed a trend where monosomies became significantly less prevalent at the more advanced developmental stages and trisomies and (hypo-)triploidies followed the opposite tendency. However, the incidence of segmental errors remained consistent across all the developmental stages investigated.

Unsurprisingly, as was reported previously [11], we observed a link between embryo morphology and aneuploidy. The overall aneuploidy incidence in embryos classed as having grade 2 “good” morphology (n = 640) was 19.7%, as opposed to just 10.7% in embryos classed as grade 1 “excellent” (n = 1073), resulting in a significant difference (*p* < 0.0001). The different aneuploidy classes were also unequally represented between the two embryo grades, suggesting that embryo morphology is associated with not just the presence but also the type of aneuploidy. Interestingly, trisomies where overrepresented in the embryos of grade 1 where they affected 44.3% of the aneuploid embryos (n = 51/115), as compared to 25.4% of the aneuploid grade 2 embryos (*p* = 0.002). Conversely, monosomies tended to affect the aneuploid grade 2 embryos more prominently with an incidence of 50% (n = 63/126), as compared to 38.3% in the aneuploid grade 1 embryos (n = 44/115); however, this difference was not significant (*p* = 0.067). The other aneuploidy classes were equally distributed across the different embryo grades (as per Figure 3B).

Notably, following ET, grade 1 embryos achieved better pregnancy rates than grade 2 embryos (55.4% versus 46.6%, *p* = 0.0004). Unexpectedly, however, live births per embryo transferred were statistically similar between grade 1 and grade 2 embryos (41.8% versus 39.4%, *p* = 0.33), suggesting that morphology alone may not be a faithful indicator of post transfer developmental potential.

### 3.6. Male Embryos (XY) Achieve Higher Morphology Scores But Are Disproportionately Affected by Aneuploidy

**Figure 4:**
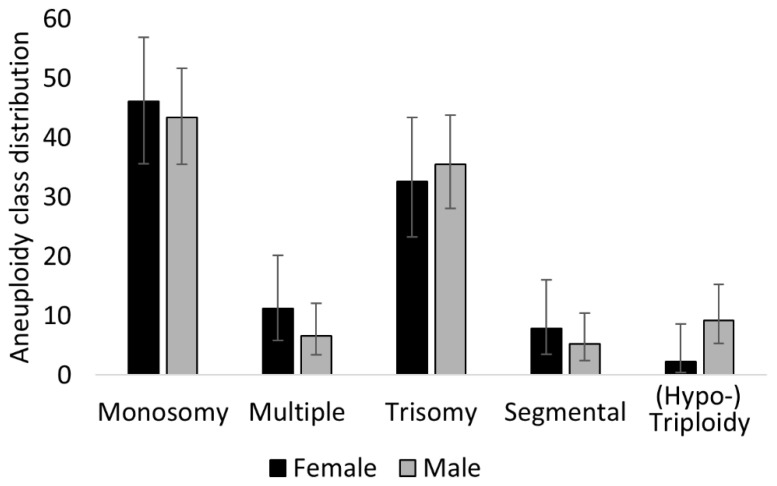
Relationship between embryo sex and aneuploidy. Although, overall, aneuploidy incidence was higher in XY embryos as compared to XX embryos, the different aneuploidy classes were equally distributed between them. Data presented as percentage (%) with 95% CI.

This database contained a significant excess of XY embryos as compared to XX embryos (n = 925 males versus n = 788 female, *p* = 0.0009), as male embryos were more often required for transfer by the breeder. Interestingly, XY embryos tended to have better morphology, with 67.1% (n = 621/925) of them being assigned to grade 1, as compared to just 57.4% (n = 452/799) of XX embryos (*p* < 0.0001). Additionally, we discovered that XY embryos had a higher incidence (*p* = 0.002) of chromosomal errors (16.4%, n = 152/925) as compared to XX embryos (11.3%, n = 89/788). On the other hand, the different aneuploidy classes were equally represented between sexes, as reported in Figure 4. This represents a novel observation in cattle, which is curiously at odds with what was just discussed in terms of embryo morphology and its relationship with sex.

### 3.7. Incidence of Errors by Chromosome

**Figure 5:**
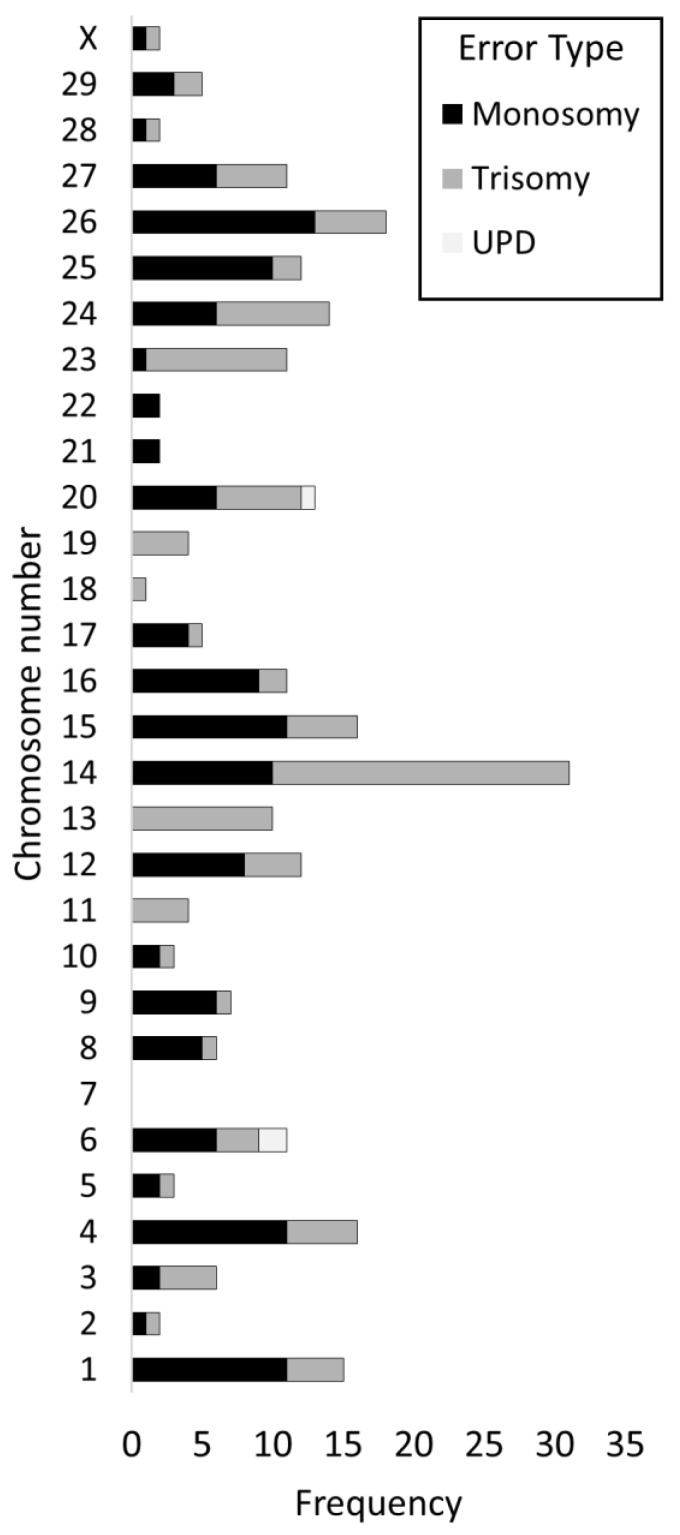
Whole chromosome error incidence by chromosome. Individual chromosomes were affected by aneuploidy at different rates, with specific chromosomes displaying a higher or lower incidence than expected. For example, monosomy and trisomy affected chromosome 14 at a much higher rate, whilst errors involving chromosome 7 were not detected in this database. N = 255 whole chromosome errors.

When triploidy/hypotriploidy events were excluded (a single event such as polyspermy which affects many chromosomes simultaneously), the chromosome specific incidence of aneuploidy seemed to vary considerably. Chromosomes 14 and 26 suffered the highest error frequencies (n = 31/255 and n = 18/255, respectively), where chromosome 14 had the highest incidence of trisomies (n = 21/31) and chromosome 26 had a greater incidence of monosomies (n = 13/18). On the other hand, chromosome 7 did not show any type of error across the entire database. Of note, chromosomes 11, 13, 18, and 19 only showed trisomies, whilst chromosomes 21 and 22 showed only monosomies. A comprehensive summary of these results is given in Figure 5.

### 3.8. Chromosomal Loss in Hypotriploidy

**Figure 6:**
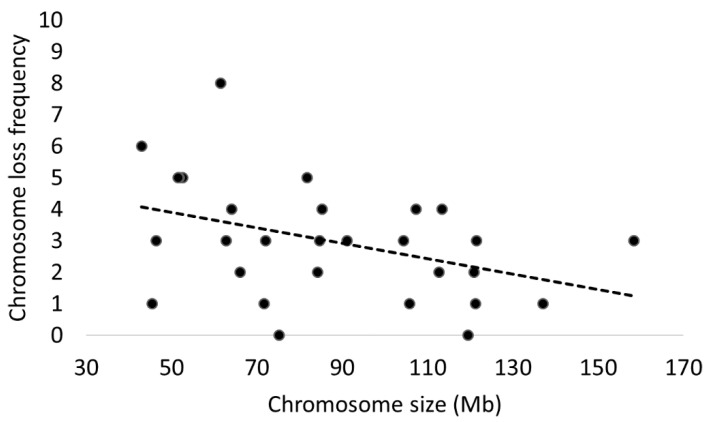
Frequency of chromosome loss in hypotriploidy. A significant trend was present, suggesting smaller chromosomes are more often lost in hypotriploidy (*p* = 0.016), even though the goodness of fit of this model was only modest (R^2^ = 0.166).

By analysing 15 cases of hypotriploidy (embryos with a less than complete triploid set of chromosomes), we observed a higher frequency of chromosome loss on chromosome 22 (n = 8/15) followed by chromosome 25 (n = 6/15), while chromosomes 6 and 17 did not show any chromosome loss. When chromosomes were arranged by size (measured in Mb), a significant trend emerged suggesting that smaller chromosomes are more commonly affected by chromosome loss in hypotriploidy (*p* = 0.016), perhaps highlighting a mechanistic link between these two parameters. A visual representation of this analysis is proposed in Figure 6.

## 4. Discussion

The results presented herein indicate that PGT-A is an effective selection strategy in cattle IVP, where embryos diagnosed as euploid are over 10 times more likely to implant than those diagnosed as aneuploid. Moreover, the exclusive use of embryos diagnosed as euploid significantly improves pregnancy and live births rates and would decrease the number of ETs required to obtain a live birth from 2.45 ETs/live birth to 2.14 ETs/live birth. This could potentially lead to significant financial savings as well as an improvement in efficiencies with environmental benefits (fewer dams needed on the ground to accept embryo transfers). The proposed strategy benefits from a high PPV (98%) and seems particularly effective in the context of the modern cattle industry, which is increasingly reliant on IVP as a breeding technology [23] and has started implementing routine embryo biopsy and SNP genotyping for the imputation of GEBVs. As demonstrated here, in doing so breeders are already accumulating sufficient information for a comprehensive screen of chromosome abnormalities in their embryos, in addition to undertaking genomic selection.

### 4.1. Relationship between Chromosomal Abnormality and Embryo Morphology

The overall incidence of chromosome abnormalities detected in this study (14.1%) is considerably lower than previously reported in cattle (30% and higher, [9,10,11]). However, we suggest this lower figure is actually a substantial underestimation of the true overall incidence of chromosomal abnormalities following bovine IVP. This is due to the fact that this database only contains embryos pre-selected under strict morphological criteria by their breeder to maximise embryo biopsy survival, cryosurvival, and later live birth rates. Indeed, a link between embryo morphology and aneuploidy incidence has already been demonstrated [11,24], and this association is supported in our present work, especially for embryos biopsied at earlier developmental stages. Nonetheless, our findings strongly suggest an added benefit for PGT-A in addition to morphology screening in IVP, as we have shown that chromosomally abnormal embryos are still able to achieve the highest morphology scores. Furthermore, we present strong evidence that aneuploidy rates change significantly with the embryonic stage, with embryos still classified as blastocyst (stages 5 to 9) displaying widely different results. As such, we suggest that any aneuploidy incidence comparison between multiple studies should carefully consider the embryo developmental stage.

### 4.2. Certain Chromosomes Are Preferentially Affected by Aneuploidy

Whilst previous work by this group has already presented a breakdown of chromosome specific errors and aneuploidy classes affecting cattle embryos [10,11], the present study is more comprehensive in this respect thanks to the considerably larger sample size. We were able to highlight how certain cattle chromosomes are much more prone to aneuploidy (chromosome 14, in particular, followed by chromosome 26), while others (like chromosomes 2, 7, and 18) present very few or no errors. The distribution of errors between chromosomes challenges, at least in cattle, the observation that there is a reverse correlation between chromosome size and incidence of aneuploidy [25]. Instead, it suggests that chromosome specific properties might be worthy of consideration. However, a bias introduced by a different degree of lethality associated with aneuploidy affecting specific chromosomes cannot be discounted, since embryos were tested only at the blastocyst stage. We could imagine, for example, that a trisomy of chromosome 7 might almost always result in early developmental failure or severely compromised blastocyst morphology and thus was never detected in this study. This latter hypothesis is also supported by the presented evidence on chromosomal loss in hypotriploidy, where smaller chromosomes were indeed lost significantly more.

### 4.3. Trisomic Embryos Survive Longer than Embryos Carrying a Monosomy

Monosomies were the most frequent abnormality observed in this database followed by trisomies, a finding that compares well with recent trials in humans [6]. The fact that the number of trisomies is less than monosomies could be the consequence of a trisomy rescue producing euploid cells [26,27]. Nevertheless, we were also able to demonstrate that the relative frequency of certain aneuploidy types changes dramatically with the embryonic stage, with advanced embryos being more commonly affected by trisomies. This finding may not seem surprising when one considers that embryos tolerate trisomies better than monosomies. The extended culture of human embryos also confirmed that trisomies and duplications were more frequent than monosomies and deletions at later stages [28].

### 4.4. Segmental and Mosaic Errors

Segmental aneuploidies were also detected in this database. The transfer of embryos affected by these errors resulted in just one live birth (out of 21 transfers). A thorough investigation of the impact of this class of chromosomal abnormality remains challenging and may require a more substantial database. We detected mosaicism in at least a quarter of the embryos identified as chromosomally abnormal, but there was no difference between mosaic and non-mosaic embryos in terms of pregnancy and live birth. However, this result should be interpreted with caution given the very limited number of chromosomally abnormal embryos resulting in a live birth, and that SNP chip data (e.g., compared to next generation sequencing data) are limited in their ability to detect mosaicism. As such, the study of mosaicism in cattle IVP would certainly merit further exploration in the future.

### 4.5. The Origin of Aneuploidy in Cattle IVP

Interestingly, the vast majority of whole chromosome errors arose in the maternal germline and, in particular, the Gabriel–Griffin plot analysis suggested that 73.5% of trisomies occurred during meiosis I in the oocyte. Although reconstructing the meiotic origin of monosomy is not possible by Gabriel–Griffin plots, it could be speculated that a similar proportion of these errors would also have arisen in meiosis I. This analysis therefore confirmed that, in cattle oocytes, meiosis I appears to be more prone to chromosomal errors than meiosis II, in concordance with similar observations previously performed on human gametes [19,29]. Human oocytes are not routinely matured in vitro (contrarily to cattle); however, current evidence seems to suggest meiosis I is a critical step for aneuploidy in both organisms.

Previous work in the bovine model has identified significantly less aneuploidy in embryos derived from in vivo matured rather than IVM oocytes [30]. Within such a context, our results provide additional support for the findings of a recent study [11] which indicated that in vitro maturation of cattle oocytes (but not ovarian stimulation) is responsible for a significant proportion of the aneuploidy errors seen in IVP. The predominance of maternal meiotic errors in embryos from young donor females (where maternal age would not be a concern) implicates IVM as the step at which most chromosomal abnormalities arise, since meiosis is still in progress during oocyte culture.

### 4.6. Differences between XX and XY Embryos

The incidence of aneuploidy differed significantly between XX and XY embryos, with the male embryos being more severely affected. To the best of our knowledge, this is a novel finding and one which is in contrast to evidence collected in human IVF where a similar skew has not been observed [31,32]. Curiously, XY embryos tended to achieve better morphology grades; therefore, an IVP system relying solely on embryo morphology as a selection criterion may lead to an undesirable increase in the birth of male calves and may inadvertently contribute to the overall aneuploidy incidence in IVP (due to male embryos being more severely affected).

### 4.7. The Wider Context

PGT-A is a controversial subject in human reproductive medicine [8]. While around 70 retrospective analyses of single and multi-centre studies attest to its efficacy, randomised controlled trials returned mixed results, with only the older age group (>35) showing consistently convincing beneficial effects [8]. The demonstrable physiological similarities with the human system make cattle a proxy for the study of human IVF. In particular, the bovine model we have investigated has the advantage that embryos were already committed to be biopsied and screened for a commercial necessity, removing a source of variability (the biopsy) with the breeder accepting any toll (however minor) such procedure might have on each embryo [7].

Nonetheless, it is important to note that in this work, as it is often routine in cattle, oocytes were in vitro matured before fertilization. However, IVM has found a much more limited use in human IVF. It has clearly been shown that embryos derived from IVM oocytes suffer from a higher incidence of chromosomal errors as compared to in vivo derived embryos [11,30]. As such, while a direct comparison between our data and PGT-A efficacy in clinical practice might be inappropriate, the bovine model can likely be useful for studying the impact of aneuploidy (of meiotic origin or otherwise) on pregnancy outcomes.

## 5. Conclusions

Overall, the data presented strongly support the use of PGT-A in cattle IVP where morphological grading of the embryos alone is not necessarily predictive of the embryo’s potential to develop to term. Deselecting aneuploid cattle embryos in this way would lead to higher pregnancy and live birth rates per embryo transferred, resulting in a more cost efficient and environmentally sustainable system thanks to the reduced need for ET surrogates. The present findings add a new model to the body of evidence reporting a positive effect of PGT-A on ET outcomes in a system like cattle IVP, which is largely unaffected by issues of advanced maternal age but where in vitro maturation is routinely performed. Interestingly, all the embryos in this study were produced and biopsied at a single centre, thus reducing the methodological variation that may affect multi-centre studies and removing any possible bias introduced by the biopsy procedure itself between tested and untested embryos. The findings reported in this retrospective analysis should be followed up by a prospective study design where embryos are transferred after PGT-A based recommendations.

## Figures and Tables

**Table 1:** SNP chips employed in this study. All chips were manufactured by Illumina (as part of their bovine range).

SNP Chip Name	Number of SNPs	Embryos Tested (n)	Parents Tested (n)
GGP Bovine HD 150k v01	138,892	379	-
GGP Bovine HD 150k v03	139,376	1241	-
GGP Bovine HD 150k v04	140,668	112	-
GGP Bovine LD v04	30,105	5	-
GGP Bovine SNP50	45,187	-	241

“-” indicates no sample of this kind was tested on the corresponding platform.

**Table 2:** Chromosomal abnormality class and frequency as identified by PGT-A. (**A**) Overview of all chromosomal errors detected, including a breakdown by error class and origin (maternal germline, paternal germline, embryonic). (**B**) Detailed overview of whole chromosome errors, providing a breakdown of error class (monosomy, trisomy, uniparental disomy (UPD)) and its origin; for trisomies, a further indication is provided to describe whether the error arose in meiosis I, in meiosis II, or during embryonic development (mitotic errors). In the case of UPDs, the origin is given as the parent passing two copies of its own chromosomes, or as the embryo in cases of a mosaic configuration.

**(A)**			**Origin**	
**Aneuploidy class**	**Overall**	**Dam**	**Sire**	**Embryo**
Segmental errors	21	2	14	5
Triploidy and hypotriploidy	16	6	10	-
Whole chromosome	255	212	16	27
Total errors	292	220	40	32
(**B**)			**Origin**	
**Whole chromosome**	**Overall**	**Dam**	**Sire**	**Embryo**
Trisomy	113	90	1	22
MI	84	83	1	-
MII	7	7	-	-
Mitotic	22	-	-	22
Monosomy	139	120	15	4
UPD	3	2	-	1

“-” indicates no events of the corresponding origin were recorded.

**Table 3:** Live birth cases from aneuploid embryos. Chr = chromosome; UPD = uniparental disomy.

Sample	Sex	Embryo Stage	Embryo Grade	Diagnosis	Chr	Mosaic	% Mosaicism
1536	M	6	1	Maternal Monosomy	4	-	-
816	F	5	2	Maternal Monosomy	22	-	-
1173	M	7	1	Maternal Trisomy	4	-	-
37	M	7	1	Maternal Trisomy	26	-	-
1437	M	6	1	Maternal Trisomy	26	-	-
1127	M	6	1	Maternal Trisomy	27	-	-
1524	M	5	2	Mitotic Trisomy	6	Yes	76.0%
257	M	6	2	Segmental Deletion	17	Yes	47.3%
482	F	6	2	UPD	6	-	-
1021	M	8	1	Hypotriploidy (polyspermy)	n/a	Yes	21.7 to 41.4%
353	M	6	2	Hypotriploidy (polyspermy)	n/a	-	-
1407	M	6	2	Hypotriploidy (polyspermy)	n/a	Yes	21.0 to 47.0%

n/a = not applicable. “-” indicates the sample had a non-mosaic configuration, as such, no “% mosaicism” is calculated.

**Table 4:** Aneuploidy incidence, pregnancy, and live birth rates among embryos of different development stages. All three parameters were significantly affected by the embryo’s development stage (*p* < 0.001). Only one stage 4 embryo was present in the database and it was therefore excluded from this analysis. Data are presented as mean with 95% CI.

Embryo Stage	n	Aneuploidy Incidence (%)	Pregnancy Rate D60 (%)	Live Birth Rate (%)
5	308	24.0 (19.6–29.1)	47.7 (42.2–53.3)	35.4 (30.3–40.9)
6	786	15.0 (12.7–17.7)	50.5 (47.0–54.0)	41.0 (37.6–44.4)
7	515	9.1 (6.9–11.9)	54.9 (50.6–59.2)	42.1 (37.9–46.5)
8–9	103	1.9 (0.5–6.8)	62.1 (52.5–70.9)	49.5 (40.0–59.0)

## Data Availability

Not applicable.

## Supplementary Materials

The following are available online at https://www.mdpi.com/article/10.3390/cells10092284/s1, Table S1: Trisomy origin.xlsx, Table S2: Aneuploidy and Mosaicism.xlsx.
